# Differential Effects of High-Fish Oil and High-Lard Diets on Cells and Cytokines Involved in the Inflammatory Process in Rat Insulin-Sensitive Tissues

**DOI:** 10.3390/ijms15023040

**Published:** 2014-02-20

**Authors:** Lillà Lionetti, Maria Pina Mollica, Raffaella Sica, Immacolata Donizzetti, Giorgio Gifuni, Angelica Pignalosa, Gina Cavaliere, Rosalba Putti

**Affiliations:** Department of Biology, Via Mezzocannone 8, University of Naples “Federico II”, 80134 Naples, Italy; E-Mails: lilla.lionetti@unina.it (L.L.); mariapia.mollica@unina.it (M.M.); raffaella.sica@unina.it (R.S.) imma.donizetti@hotmail.it (I.D.); giorgio.gifuni@libero.it (G.G.); angelicapignalosa@libero.it (A.P.); gina.cavaliere@libero.it (G.C.)

**Keywords:** adipokines, α-SMA, TGF-β1, MCP1, dietary fat source

## Abstract

Dietary fat sources may differentially affect the development of inflammation in insulin-sensitive tissues during chronic overfeeding. Considering the anti-inflammatory properties of ω-3 fatty acids, this study aimed to compare the effects of chronic high-fish oil and high-lard diets on obesity-related inflammation by evaluating serum and tissue adipokine levels and histological features in insulin-sensitive tissues (white adipose tissue, skeletal muscle and liver). As expected, a high-lard diet induced systemic and peripheral inflammation and insulin resistance. Conversely, compared with a high-lard diet, a high-fish oil diet resulted in a lower degree of systemic inflammation and insulin resistance that were associated with a lower adipocyte diameter as well as lower immunoreactivity for transforming growth factor β 1 (TGFβ1) in white adipose tissue. A high-fish oil diet also resulted in a lower ectopic lipid depot, inflammation degree and insulin resistance in the skeletal muscle and liver. Moreover, a high-fish oil diet attenuated hepatic stellate cell activation and fibrogenesis in the liver, as indicated by the smooth muscle α-actin (α-SMA) and TGFβ1 levels. The replacement of lard (saturated fatty acids) with fish oil (ω-3 fatty acids) in chronic high-fat feeding attenuated the development of systemic and tissue inflammation.

## 1. Introduction

Obesity is associated with a state of chronic, low-grade inflammation in which crosstalk occurs at the intra-organ and inter-organ levels [1–3]. The intra-organ crosstalk is due to the reciprocal interplay between parenchymal and stromal vascular cells, whereas the inter-organ crosstalk is due to metabolic and endocrine mediators, which, in turn, stimulate new interactions between parenchymal and non-parenchymal cells in target organs. Chronic overfeeding with a high-fat diet causes an increase in white adipose depots as well as ectopic lipid accumulation in non–adipose tissues such as the liver and skeletal muscle and is associated with the development of tissue insulin resistance (IR) and a pro-inflammatory state [1,2,4].

Given that the amount of dietary fat intake has long been proposed as a causative factor in the inflammatory process that is associated with obesity, the quality of dietary fat may also play a key role in these crosstalk mechanisms. Diets rich in saturated fatty acids (SFAs) have been associated with an increased risk for obesity, IR and atherosclerosis, whereas diets containing long chain ω-3 polyunsaturated fatty acids (PUFAs) from fish oil have been shown in many studies to protect against these metabolic diseases [5–8]. Several previous studies have also indicated that ω-3 PUFAs possess anti-inflammatory properties that prevent and reverse the development of insulin resistance in mice fed a high-fat diet in an adiponectin-dependent manner [9–16].

Most of the studies on the positive effects of ω-3 PUFAs were designed to assess the effects of ω-3 PUFA supplementation with high-fat diet *in vivo* or to assess their effects *in vitro*. To assess the effect of the quality of dietary fat (SFAs compared with ω-3 PUFAs) on the development of inflammation *in vivo* in insulin-sensitive tissues, we used a chronic overfed animal model in which the two different high-fat diets provided an identical amount of fat (40% of energy from fat) but differed in the type of fat used, *i.e.*, fish oil (primarily ω-3 PUFAs) compared with lard (primarily SFAs). Preliminary data obtained in our lab suggested that high fish oil feeding elicited a lower body weight gain compared to high lard feeding due to an increased total body energy expenditure associated with an increase in lipid oxidation and a decrease in energy efficiency in isolated liver and skeletal muscle mitochondria [17,18]. The goal of the present study was to compare the *in vivo* effects of high-fish oil (F) and high-lard diets (L) on the inflammatory process and development of IR at both the systemic and the tissue levels in the three primary insulin-sensitive tissues, namely white adipose tissue (WAT), skeletal muscle and liver. In particular, we examined the effect of the two different diets on the following criteria: (1) adipocyte size, monocyte chemotactic protein 1 (MCP1) and transforming growth factor β 1 (TGFβ1) levels, macrophage activation and insulin resistance markers in WAT; (2) ectopic lipid accumulation and inflammatory and insulin resistance markers in skeletal muscle; and (3) ectopic lipid accumulation, Kupffer cell and hepatic stellate cell (HSC) activation as well as the possible onset of fibrogenesis in the liver. The results of the present study agreed with previous results regarding the anti-inflammatory properties of ω-3 fatty acids, providing a comprehensive view of the effects of the quality of dietary fat in a chronic high-fat diet on the three insulin-sensitive tissues that are involved in inflammation and IR.

## 2. Results and Discussion

### 2.1. Body Weight Gain and Energy Intake

At the end of the experimental period, the high-lard fed (L) rats demonstrated the highest body weight gain per week among the groups (Table 1). The high-fish oil fed (F) rats demonstrated an increase in food intake per week that was similar to that found in the L rats (Table 1), but the body weight gain was lower in F than in L rats. The feeding efficiency, which is expressed as weight gain (grams)/food intake (grams) × 100, was higher in the L compared with the F rats. In addition, retroperitoneal and epididymal adipose tissue weight were lower in F than in L rats. Thus, the replacement of lard (primarily saturated fatty acids) with fish oil (primarily ω-3 fatty acids) in a high-fat diet during chronic overfeeding attenuated the development of obesity in accordance with previous studies [5,7].

### 2.2. Serum Metabolite, Hormone and Adipokine Levels

**Figure 1.**
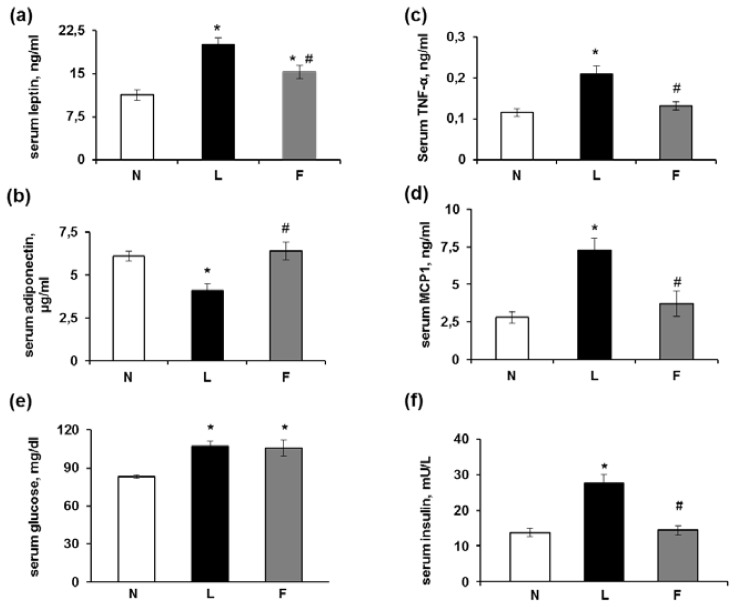

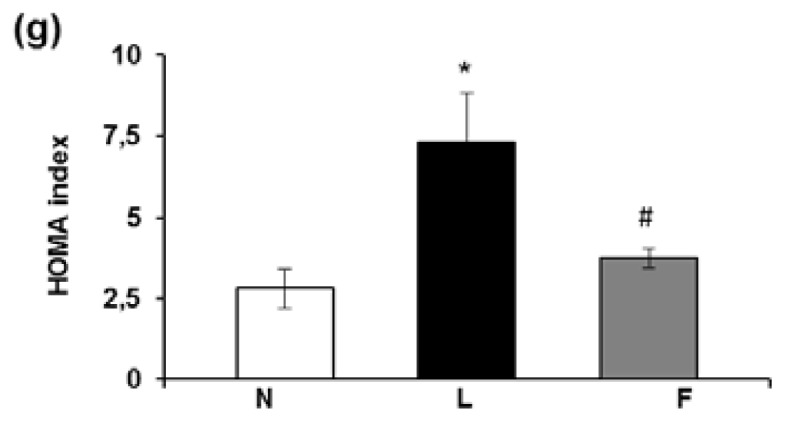
Serum adipokines. (**a**,**b**) Serum leptin and adiponectin levels; (**c**,**d**) Serum TNF-α and MCP1 levels and (**e**,**f**) Serum glucose and insulin levels; (**g**) HOMA index. The data represent the means ± SE for 8 rats in each experimental group. * *p* < 0.05 compared with N rats; and ^#^ *p* < 0.05 compared with L rats.

As shown in Figure 1, the serum leptin levels (Figure 1a) were higher in the L and F rats compared with the standard diet fed (N) rats but the F rats demonstrated a lower level compared with the L rats. The L rats demonstrated a lower serum adiponectin level compared with the N rats, whereas the F rats demonstrated normal serum adiponectin levels (Figure 1b). Figure 1 also shows the serum TNF-α (Figure 1c) and MCP1 (Figure 1d) levels, which markedly increased in the L rats compared with the N rats. The F rats also demonstrated increased serum concentrations of TNF-α and MCP1 compared with those of the N group, but this increase was significantly lower compared with the increase found in the L rats.

The serum glucose levels significantly increased in both the L and F rats compared with the N rats (Figure 1e). However, the L rats demonstrated significantly higher insulin levels compared with both the N and F rats (Figure 1f). Thus, the homeostatic model assessment HOMA index (calculated as (Glucose (mg/dL) × Insulin (mU/L)]/405) was significantly higher in the L rats compared with the N and F rats (Figure 1g).

Our results are consistent with those of previous studies using high-fat-induced obesity models [1–3] in which an increase in serum inflammatory markers (MCP1, TNFα) and leptin levels were reported, as was a decrease in adiponectin that was associated with an increased HOMA index. However, our results on the HOMA index and leptin levels in the F rats were consistent with previous studies that indicated that eicosapentaenoic acid (EPA) minimized saturated fat-induced IR in C57BL/6J mice [16] and increased adipocyte leptin secretion *in vitro* [19,20]. In contrast, Reseland *et al.* [21] reported that dietary *n*-3 PUFAs decreased leptin mRNA expression *in vivo* and *in vitro*. The lower increase in circulating leptin levels found in the F compared with L rats may be secondary to decreases in WAT mass, as suggested by the reduced body weight gain.

The effects of ω-3 fatty acids (such as EPA and docosahexaenoic acid (DHA)) on the prevention of excess weight gain and the development of IR that is associated with HF feeding are thought to be mediated by adiponectin [14,16]. Accordingly, an increase in adiponectin in the adipose tissue of mice fed a high-fat diet rich in ω-3 FAs (such as EPA and DHA [10]) has been reported in the adipose tissue of ob/ob mice that received an ω-3 PUFA-enriched diet [6] and in cultured human adipocytes [22] in response to EPA. Our results that demonstrate the maintenance of normal serum adiponectin levels in F rats compared with L rats (in which these levels are lowered) are consistent with the results of Kalupahana *et al.* [16], who reported comparable serum adiponectin levels in mice fed a low-fat or a high saturated fat + EPA diet and decreased adiponectin levels in rats fed a high-saturated fat diet. The maintenance of serum adiponectin levels may play a key role in the prevention of the development of IR, as suggested by Kalupahana *et al.* [10,16]. The increased levels of adiponectin in response to fish oil appear to be due to ω-3 PUFAs, which may incorporate into the membrane phospholipid fraction of adipocytes in rats fed a fish oil diet for six weeks [23] and thus exert their beneficial influence. The adiponectin levels were related to the levels of peroxisome proliferator-activated receptors (PPARs), as discussed below.

### 2.3. eWAT Histology and Immunohistochemistry

**Figure 2.**
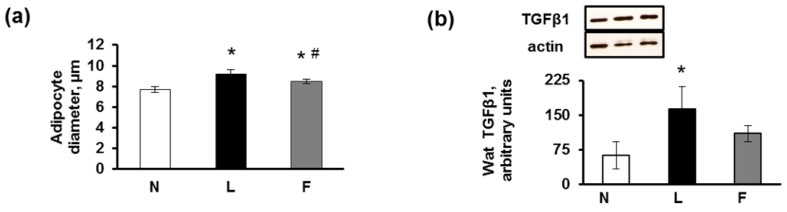

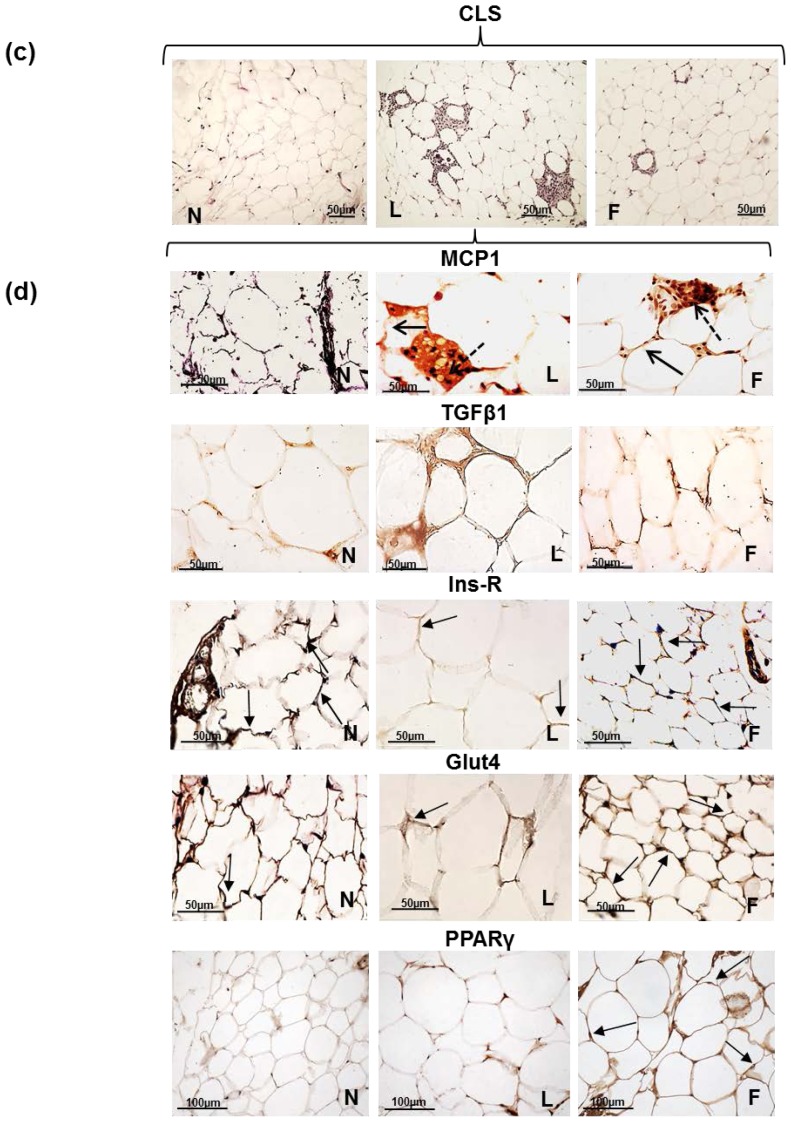
(**a**) Adipocyte diameter: The mean diameter of the L adipocytes was significantly higher compared with that of the F group. The mean adipocyte diameters of both the L and the F rats were significantly higher than that of the N group. The data represent the means ± SE (100 cells/animal, 8 animals/group). * *p* < 0.05 *vs.* N rats; and ^#^ *p* < 0.05 *vs.* L rats; (**b**) Representative Western blot analysis of TGFβ1 and corresponding densitometric analysis on the relative protein levels. The data represent the means ± SE. * *p* < 0.05 *vs.* N rats; (**c**) Histological sections of rats fed a control diet (N), high-lard diet (L) or high-fish oil diet (F) that indicate the presence and relative abundance of crown-like structures (CLSs) around hypertrophic adipocytes. No CLSs were observed in the N rats; (**d**) Immunostaining of histological sections of the N, L and F rats. MCP1: No immunostaining for MCP1 was observed in the N rats. Immunoreactivity was present in CLSs (dashed arrow) and in the rim of the cytoplasm of adipocytes in the L and F rats (arrow). TGFβ1: The immunostaining was weak in the N section, whereas the L section demonstrated the strongest immunostaining among the groups. InsR (insulin receptor): Following insulin administration (15 min after an i.p. injection of insulin (homologue rapid-acting, 10 units/kg body wt; Novartis)), the N and F eWAT sections exhibited strong immunoreactivity in some areas (arrows), whereas weak immunoreactivity was localised in the L adipocytes (arrows). Glut4: After the insulin injection, the immunostaining for the glucose transporter Glut4 demonstrated an identical distribution as that of InsR and was evident in N and F (arrows) sections, whereas the L section demonstrated weak Glut4 immunostaining that occurred in areas with apparent stromal-vascular cell localization (arrows). PPARγ: Immunoreactivity was observed in the adipocyte cytoplasm and in the vascular stromal cells in all groups of rats. In the F rats, we found the highest immunolabelling of both adipocyte cytoplasm and nuclei, which was consistent with the translocation of PPAR into the nuclei (arrows).

In eWAT, the mean diameter of the adipocytes was lower in the N and F compared with the L rats, which is consistent with the lower body weight found in the N and F rats (Figure 2a). Consequently, although the eWAT of the N group exhibited a normal appearance without signs of inflammation, the L rats developed the largest hypertrophic adipocytes surrounded by a higher number of crown-like structures (CLSs) than in the F rats, indicating inflammation marked by the presence of macrophages/monocytes surrounding the hypertrophic dying adipocytes (Figure 2c). This finding is consistent with previous results that found that macrophage numbers in adipose tissue increase with obesity, in which they apparently function to scavenge moribund adipocytes [24,25].

The immunohistochemical staining for MCP1, which is the small proinflammatory chemokine that promotes macrophage mobilization from bone marrow into tissues [26], revealed no or extremely weak positivity in the N rats, whereas a strong positivity was observed in the L and F rats, which was primarily localized on the CLSs (Figure 2d). The CLSs demonstrated the highest immunostaining for MCP1, both on adipocytes (confirming that MCP1 was secreted by adipocytes themselves, as found by Kanda *et al.* [27]) and on macrophage aggregates, from which MCP1 is predominantly produced [28]. Consistent with our results, increased expression levels of MCP1 have also been reported in the WAT of genetically obese mice or mice fed a high-fat diet [26,29]. In F rats the CLSs were present although to a smaller extent than L group, as a consequence MCP1 immunostaining was also evident in F group. This result probably was due to the high caloric diet of these rats, which leads to a certain degree of inflammation even if in a lower degree compared to L group.

In our experimental rats, we also detected a higher TGFβ1 protein content and immunostaining in the L compared with the F adipocytes and macrophages (Figure 2b–d). Our results corroborated those of previous studies that indicated that TGFβ1 mRNA and protein levels were increased in the adipose tissue of genetically obese mice compared with lean mice and in TNFα-stimulated cultured adipocytes [30]. TGFβ1 plays an important role in obesity, inflammation and metabolic syndrome (MetS). Sciarretta *et al.* [31] demonstrated that the serum levels of inflammatory markers, including TNF-α and TGFβ1, in hypertensive patients with MetS were significantly higher than those in patients without MetS. Previous studies have demonstrated that macrophage infiltration causes IR [25,29] and that TGFβ1 is involved in this mechanism [32]. Elevated TGFβ1 signalling evokes adipocyte hypertrophy, hyperplasia and elevated pro-inflammatory adipokine production, which in turn interfere with insulin signalling and promote the recruitment of “classically activated” M1 macrophages to the WAT [32]. The phenotypic switching of macrophages from an anti-inflammatory “alternatively activated” M2 to a more pro-inflammatory M1 form [33,34] was primarily indicated in the L rats by their immunoreactivity to different inflammatory adipokines, such as MCP1 and TGFβ1.

We then analysed by immunohistochemical analysis the presence of InsR and GLUT4 after insulin stimulation and the results showed a faint immunostaining of (ii)-L rats for InsR, whereas no apparent differences were observed between the F and N rats. In particular, the immunoreactivity for InsR in eWAT tissues from (ii) rats (after insulin injection) was primarily localized along the plasma membrane and the rim of the cytoplasm surrounding the lipid droplet of adipocytes; this was clearly evident in the N and F groups but was weak in the L group (Figure 2d). An identical immunoreactivity distribution was observed for Glut4 (Figure 2d). These immunohistochemical observations may suggest an impairment of insulin signaling in the L compared with F rats, even if further analysis of markers of insulin signaling pathway are needed to confirm it. However, this suggestion is consistent with previous studies that suggest a role for dietary fat in the onset of inflammation in adipose tissue, in which ω-3 PUFAs consistently prevent the development of IR that is associated with a high-fat diet in rodents [13]. Moreover, EPA increased Glut4 expression in both adipose tissue and skeletal muscle [35], and EPA and DHA may help delay the progression of MetS to type 2 diabetes [36].

Ω-3 PUFAs (EPA and DHA) biosynthesized lipid-mediators, resolvins and protectins, that play pivotal roles in resolution of inflammation [6]. These molecules are not immunosuppressive, but have potent protective and anti-inflammatory properties by activating specific mechanisms to promote homeostasis. Because resolvins upregulate PPARγ expression in adipose tissue and EPA and DHA are endogenous ligands for PPARs, which may mediate the lipid-lowering properties of fish oil [6,37], we performed immunostaining for PPARγ. The results indicated that PPARγ was localized in the narrow rim of the cytoplasm of adipocytes, in stromal-vascular cells and in only some nuclei in all three groups of rats. The strongest nuclear labelling was found in the F rats, which indicated that the translocation of PPARγ into the nuclei had occurred. Our findings are consistent with previous studies that reported that ω-3 fatty acids and their metabolites are potent PPARs agonists, which activate PPARs and allow their translocation into adipocyte nuclei. Moreover, PPARγ has been found to be necessary for adipocyte differentiation and for lipid storage [38].

In addition to the interaction between fish oil and PPARs in the regulation of glucose and lipid metabolism in the liver [6], PPARγ plays a key role in the metabolism of WAT, and its effects are mediated via adiponectin, as an increase in adiponectin was completely blocked via the administration of a PPARγ inhibitor [39]. In our study, the adiponectin level was increased in the F group, which is consistent with the effects of fish oil on PPARγ. However, the mechanisms through which fish oil and other PPARγ agonists increase adiponectin secretion remain under investigation.

### 2.4. Skeletal Muscle Fibre Histology and Immunohistochemistry

**Figure 3.**
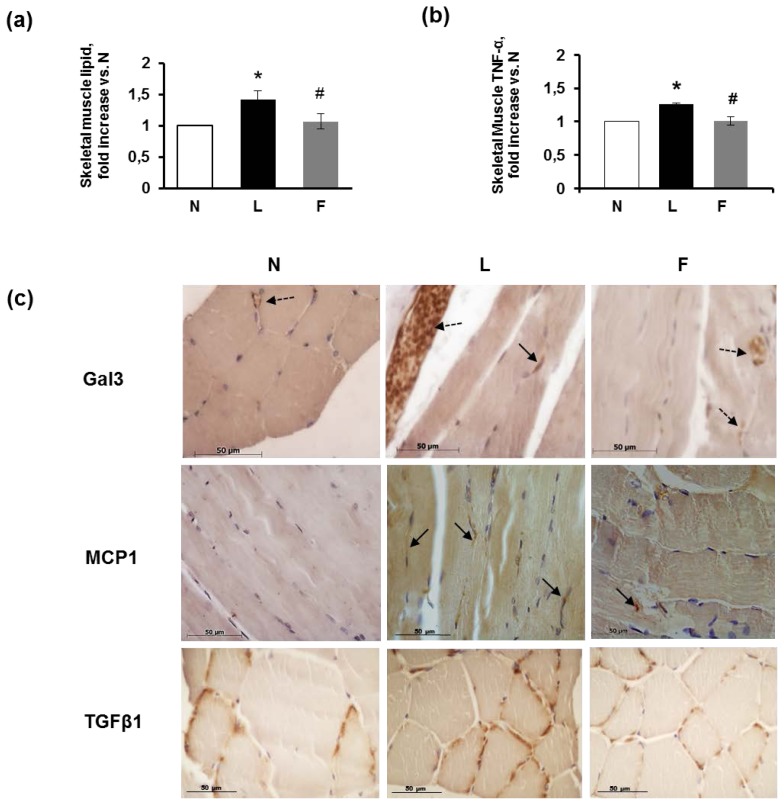
Skeletal muscle fibre analysis in rats fed a control diet (N), high-lard diet (L) or high-fish oil diet (F). (**a**) Lipid content in the skeletal muscle; (**b**) TNF-α levels in skeletal muscle; (**a**,**b**) The data are reported as fold increase *vs.* N rats (*N* = 1) and represent the means ± SE for 8 rats in each experimental group. * *p* < 0.05 compared with N rats; and ^#^ *p* < 0.05 compared with L rats; (**c**) Immunostaining in the N, L and F histological sections. Gal3: In the N rats, the immunostaining for this lectin was observed only in the endothelial cells of blood vessels (dashed arrow); in L, positive cells for Gal3 was localised near muscle fibres (arrow). Notably, the blood vessels of the endomysium that service the muscle fibres were filled with Gal3-positive cells, suggesting a switch from M2 to M1 macrophages (dashed arrow). In the F group muscle fibres, several positive cells were found in the blood vessels (dashed arrows). MCP1: N fibres did not demonstrate immunostaining for this cytokine, in contrast to the L and F muscle fibres. Arrows indicate cells (likely M1 macrophages) positive for MCP1. TGFβ1: Cells positive for TGFβ1 were found in all N, L and F sections and were localised around the muscle fibres; in particular, L fibres presented stronger labelling.

The *gastrocnemius* skeletal fibres did not exhibit a consistent lipid depot, but the L rats demonstrated the highest lipid content, as determined using Folch’s method [40] (Figure 3a). Regarding the inflammatory markers, the level of TNF-α was significantly increased in the L rats compared with the N rats, whereas the F rats demonstrated TNF-α concentrations similar to those found in the controls (Figure 3b). The immunohistochemical staining for galectin3 (Gal3), which is a lectin expressed by activated macrophages that mediates macrophage chemotactic, phagocytic and inflammatory responses [41], localized to immunoreactive cells in blood vessels (monocytes/macrophages) and to the endothelial cells of blood vessels of the endomysium in all groups of rats but to a different extent in these groups (Figure 3c). Numerous Gal3-immunoreactive cells were found in the L rats. In the F rats, a moderate number of Gal3-immunoreactive cells in blood vessels were observed. The N rats exhibited very weak Gal3 immunoreactivity (Figure 3c, Gal3).

The strong infiltration of cells positive for Gal3 in the L rats was consistent with findings by Li *et al.* [42] who demonstrated that, in HFD mice, M1 macrophages, but not M2 cells, expressed a higher intensity of Gal3 than normal mice macrophages. We suggest that the strong presence of Gal3-immunoreactive macrophages in our L rats may be due to phenotypic switching from M2 to inflammatory M1 macrophages. The M1 population demonstrates a positive correlation with IR and dominates in states of overfeeding by targeting FFA-mediated increases in pro-inflammatory responses [43,44]. However, in our treatment, the presence of on-going inflammation may be confirmed by MCP1-immunoreactive macrophages, which are more evident in the muscle fibres of the L rats. The immunostaining for MCP1 was very faint in the N group indicating few immunoreactive cells, whereas a greater number of macrophages were labelled in the L group than the F group (Figure 3c, MCP1).

In addition, TGFβ1 immunoreactivity was present in all groups of rats but was higher in the L group than in the F and N groups, which is consistent with the greater number of activated macrophages (Gal3-immunoreactive) found in the L group (Figure 3c), TGFβ1).

Preliminary data obtained in our lab suggested that high lard *vs.* high fish oil feeding exhibited a higher skeletal muscle insulin resistance related to a lower insulin-stimulated phosphorylation of protein kinase B (PKB/AKT) [17].

**Figure 4.**
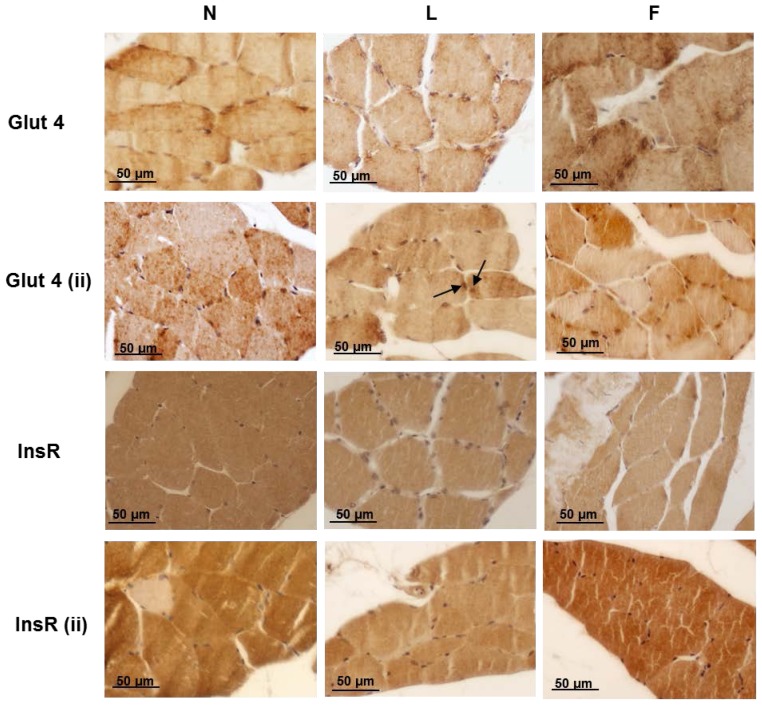
Skeletal muscle fibre immunohistochemical analysis of insulin resistance in rats fed a control diet (N), high-lard diet (L) or high-fish oil diet (F) Glut4: Without insulin administration, the immunostaining for Glut4 was evident in all groups of rats, although less in the L rats. After insulin administration (15 min after an i.p. injection of insulin), positivity for Glut4 in the N muscle fibres exhibited a diffuse punctuate distribution, suggesting translocation from perinuclear depots to the plasmalemma throughout the sarcoplasm. In the L muscle fibres, the positivity was primarily perinuclear (arrow), except in a few fibres in which the positivity was sarcoplasmic. In F muscle fibres, the immunostaining for Glut4 was massive in most of the fibres. InsR: N, F and L fibres exhibited positivity for InsR, whereas following insulin stimulation, the L fibres were only weakly positive, in contrast to the InsR positivity for N and F fibres.

To evaluate at morphological level the onset of IR in skeletal muscle, we performed an immunohistochemistry analysis in no-insulin and insulin-injected (ii) rats. The results indicated positive Glut4 immunostaining in the three groups of rats that differed in extent for each group, of which the (ii) rats demonstrated the strongest positive staining (Figure 4, Glut4). In particular, the Glut4 immunostaining in the N rats differentially labelled the muscle fibres in both the sarcoplasm and the sarcolemma. Very weak positivity for Glut4 was detected in the perinuclear depots. In the (ii)-N rats, the immunostaining for Glut4 was strong and labelled the greatest amount of the fibres, often with a diffuse and punctuate distribution, suggesting Glut4 translocation through the sarcoplasm to the plasma membrane. In the high-fat diet-treated groups, immunoreactivity to Glut4 was localised primarily around the perinuclear depot (no-insulin L fibres), whereas in the no-insulin F fibres, the distribution of Glut4 immunoreactivity resembled that of the N rats. In some fibres of the (ii)-L rats, there was partial scattered Glut4 labelling in the sarcoplasm, whereas in the (ii)-F rats, Glut4 labelling was massive (Figure 4, Glut4), suggesting insulin-stimulated translocation occurred. InsR immunoreactivity corresponded with that of Glut4, demonstrating strong labelling of the (ii)-N and (ii)-F skeletal muscle fibres but only very weak labelling of the (ii)-L muscle fibres (Figure 4, InsR). Thus, these findings in L rats suggested the onset of IR, which is consistent with the weaker immunoreactivity for pIRS1(tyr632) [45] and lower insulin-stimulated phosphorylation of AKT previously found in the L skeletal muscle [17].

### 2.5. Hepatic Steatosis

**Figure 5.**
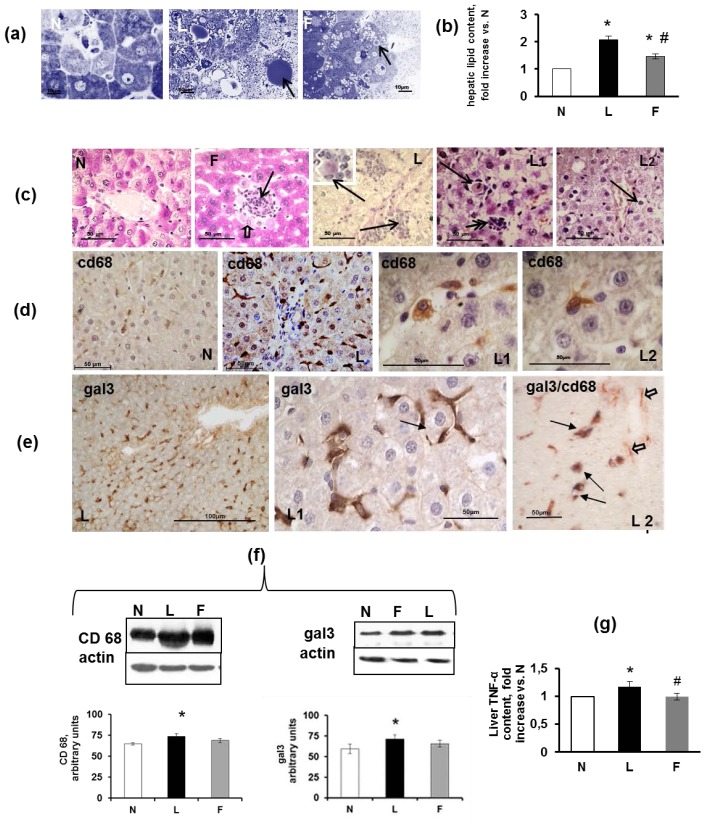
Hepatic steatosis and inflammation. (**a**) Semi-thin liver sections of rats fed a control diet (N), high-lard diet (L) or high-fish oil diet (F) that indicate the presence of lipid droplets in the L and F livers (arrows). OsO_4_ and Toluidine blue stain; (**b**) Hepatic lipid content. The data are reported as fold increase *vs.* N rats (*N* = 1) and represent the means ± SE for eight rats in each experimental group. * *p* < 0.05 compared with N rats; and ^#^ *p* < 0.05 compared with L rats; (**c**) N: a section of a control liver stained with PAS that indicates the glycogen depot. F: a section of an F liver with an infiltration of inflammatory cells (arrow) surrounded by degenerating hepatocytes, as observed by the loss of glycogen and indicated by negativity to the PAS reaction (open arrow). The other neighbouring hepatocytes contained PAS-positive glycogen. L: A section of an L liver with arrows indicating inflammatory foci along the course of the central vein. The infiltrated cells were primarily mononuclear with some neutrophils. The inset shows an apoptotic body surrounded by mononuclear and neutrophil cells at higher magnification. L1: A section of an L rat liver that shows an apoptotic body surrounded by Kupffer cells (arrows) and another cluster of phagocytic cells (double arrows). L2: A section of an L rat liver that shows ballooning hepatocytes (arrows); (**d**) CD68 immunostaining: N and L liver sections that show CD68-immunoreactive Kupffer cells localised in the liver sinusoids that demonstrate the difference in abundance of Kupffer cells between the N and L rats. L1 and L2: Higher magnification of two Kupffer cells; (**e**) Gal3 immunostaining: L and L1: Two different magnifications of an L liver that demonstrate the abundance of Gal3-immunoreactive cells (primarily macrophages) and their distribution as crown-like structures around damaged hepatocytes (arrow). Gal3/CD68: Double immunostaining for Gal3 (red) and CD68 (blue-violet). Most of the cells are double-labelled (black arrows), indicating activated Kupffer cells, whereas a few elongated cells were immunoreactive only for Gal3, suggesting that they may be trans-differentiated HSCs (open arrows); (**f**) Representative Western blot analysis of Cd68 and Gal-3 and corresponding densitometric analyses on the relative protein levels of Cd68 and Gal-3, respectively. The data represent the means ± SE. * *p* < 0.05 *vs.* N rats; (**g**) Liver TNF-α levels. The data are reported as fold increase *vs.* N rats (*N* = 1) and represent the means ± SE for 8 rats in each experimental group. * *p* < 0.05 compared with N rats; and ^#^ *p* < 0.05 compared with L rats.

The livers of the control rats did not exhibit macroscopic abnormalities. Microscopically, the livers either did not exhibit fat depots or showed small amounts of lipid droplets in the hepatocytes (Figure 5a, N). The livers of the L rats exhibited mixed steatosis (Figure 5a, L). The livers of the F rats were characterized by mixed steatosis with smaller lipid droplets than those of the L rats (Figure 5a, F). In addition, the L and F rats demonstrated a significant increase in hepatic lipid content, as assessed using Folch’s method [40] compared with N rats; however, the hepatic lipid content in the F rats was significantly lower compared with the L rats (Figure 5b). This effect may be mediated by adiponectin, which can also promote hepatic fatty acid oxidation, thus reducing lipid accumulation in the liver [46].

Regarding the development of inflammation, although the N rats did not exhibit signs of inflammation (Figure 5c, N), infiltration of inflammatory cells was present in overfed rats. This infiltration was evident as at least 1–2 inflammatory foci were observed in a same section in both F and L groups. Hepatocytes that were localized near the inflammatory foci were injured, as could be observed via their negativity to the PAS reaction (these cells lose their glycogen) (Figure 5c, F, open arrow). In the livers of the L group, the inflammatory foci were detected (Figure 5c, L, arrows); moreover, eosinophilic apoptotic bodies (Councilman-like bodies) were evident adjacent to the central vein. These bodies detached from the other cells of the plate and were surrounded by Kupffer cells with or without lymphocytes and neutrophils (Figure 5c, L-L2, arrows). In the L and F rats, glycogenated nuclei were more numerous than in the N rats. Several ballooned hepatocytes, which were characterized by cell swelling and were empty of their intracellular content, were observed in the parenchyma (Figure 5c, L2, arrow).

Regarding the histological results of the infiltration by inflammatory cells in the liver, immunohistochemical staining revealed immunoreactive cells for CD68 that identify monocyte/macrophage lineage and, therefore, liver mesenchymal Kupffer cells. The CD68-immunoreactive cells exhibited significant heterogeneity and were detected as round, rod-shaped, elongated spindle-shaped cells and, occasionally, amoeboid cells diffusely distributed in the sinusoids throughout the parenchyma. Kupffer cells were abundant in the N, L and F rats; however, in the L rats, the number of Kupffer cells appeared to increase as well as aggregate (Figure 5d) in the zones in which steatosis was present. In the livers of the N rats, immunostaining for Gal3 indicated Gal3 expression in Kupffer cells and the bile duct epithelia, demonstrating a distribution similar to that of CD68. The Gal3-immunoreactive cells of the L rats tended to aggregate to form multinucleate giant cells; additionally, the Gal3-immunoreactive cells and their protrusions formed a wide net throughout the liver parenchyma, primarily encircling injured hepatocytes (Figure 5e, L-L1). The double immunostaining for Gal-3/CD68 indicated that the most immunoreactive cells were labelled by both antibodies (Figure 5e, L2 arrows). However, it is interesting to note that some elongated cells were only Gal3-immunoreactive. These cells may be myofibroblasts (Figure 5e, L2 open arrows), demonstrating that not only Kupffer cells expressed this protein, as was also suggested by the larger number of Gal3-immunoreactive cells compared with CD68-immunoreactive cells. The cells with morphological features of HSCs, e.g., the presence of numerous lipid droplets, were labelled by this antibody, which is consistent with the finding that Gal3 expression was temporally and spatially associated with fibrosis in a rat model of reversible carbon tetrachloride (CCl4)-induced liver fibrosis [47]. Moreover, monocyte recruitment may also contribute to an increased number of Gal3-immunoreactive cells. The increased number of CD68-immunoreactive and Gal3-immunoreactive cells was confirmed using Western blot analysis, which demonstrated levels of CD68 and Gal3 that were significantly higher in the L compared with the N and F rats (Figure 5f). Consistent with this finding, the L rats demonstrated the highest level of liver TNFα, as evaluated using ELISA (Figure 5g). The lower level of TNFα found in the F rats compared with the L rats was consistent with a lower degree of hepatic IR in the F rats, as suggested by the results that indicated a higher degree of hepatic AKT phosphorylation in F than in L rats, as previously determined (unpublished data).

**Figure 6.**
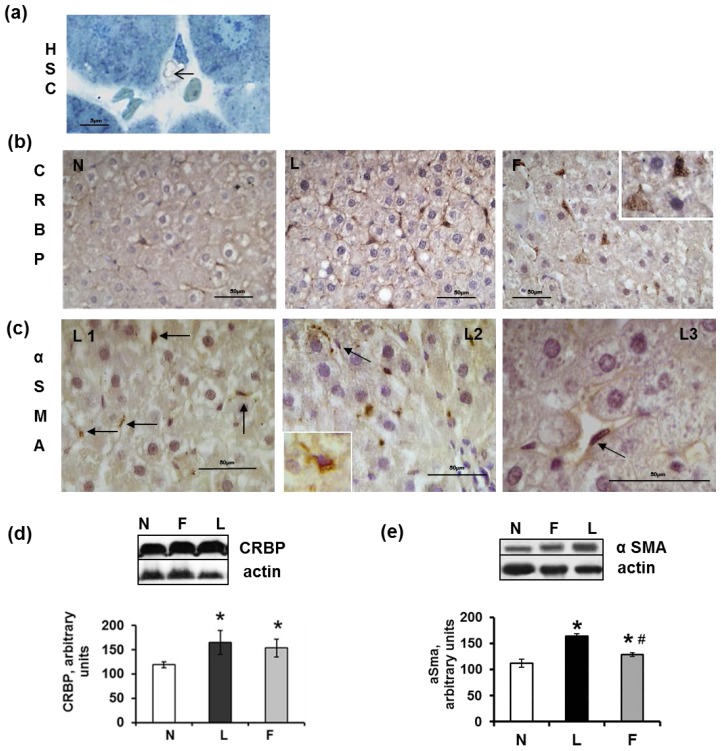
Hepatic stellate cells (HSC) in sections from rats fed a control diet (N), high-lard diet (L) or high-fish oil diet (F). (**a**) Semi-thin section of an N liver that shows the cytoplasm of an HSC that is filled with lipid droplets (arrow); (**b**) N: CRBP-immunoreactive cells (HSCs) appeared to form a network around the N hepatocytes. L: CRBP-immunoreactive cells appeared to be more numerous than those of the N rats. F: strong positivity for CRBP in HSCs. The inset shows a higher magnification of CRBP-immunoreactive HSCs that are present in the Disse space; (**c**) L1 and L2: The arrows indicate the positivity for α-SMA in elongated cells of the walls of blood vessels. L3: A higher magnification of an α-SMA-positive HSC; (**d**) Representative Western blot analysis of CRBP and corresponding densitometric analyses on the relative protein levels in the N, L and F rat livers. The data represent the means ± SE. * *p* < 0.05 *vs.* N rats; (**e**) Representative Western blot analysis of α-SMA and corresponding densitometric analyses on the relative protein levels of the N, L and F rat livers. The data represent the means ± SE. * *p* < 0.05 *vs.* N rats. ^#^ *p* < 0.05 compared with L rats.

HSCs represent an additional type of mesenchymal liver cells that are known to participate in the liver’s response to injury. These cells lie in the space of Disse that separates hepatocytes from the sinusoidal endothelium. As shown via sections, these cells appeared in close proximity to Kupffer cells. In the normal liver, quiescent HSCs possess long and thin protrusions through which these cells come into contact with endothelial cells of the sinusoidal wall. These cells represent a fat and retinol-storing phenotype (Figure 6a), which was detected via immunohistochemistry using anti-CRBP1 (cellular retinol-binding protein 1) antiserum. In contrast to the intrasinusoidal localisation of CD68-immunoreactive Kupffer cells, the CRBP1-immunoreactive HSCs underlined the sinusoids and were abundant in both the control and high-fat diet rats. A strong expression of CRBP1 was observed in HSCs, whereas a weak expression was occasionally present in some Kupffer cells (Figure 6b). The number of CRBP1-positive cells appeared to increase in the L and F rats compared with the N rats. Western blot analysis of the liver homogenates indicated a significantly increased expression of CRBP1 in the L and F compared with the N rats (Figure 6d). The increased presence of HSCs in both the L and F rats may be related to an early response to liver injury because this cell type is responsible for the wound healing that occurs during liver damage and is characterized by the excessive production and deposition of extracellular matrix (ECM) components, primarily collagen type I [48,49].

**Figure 7.**
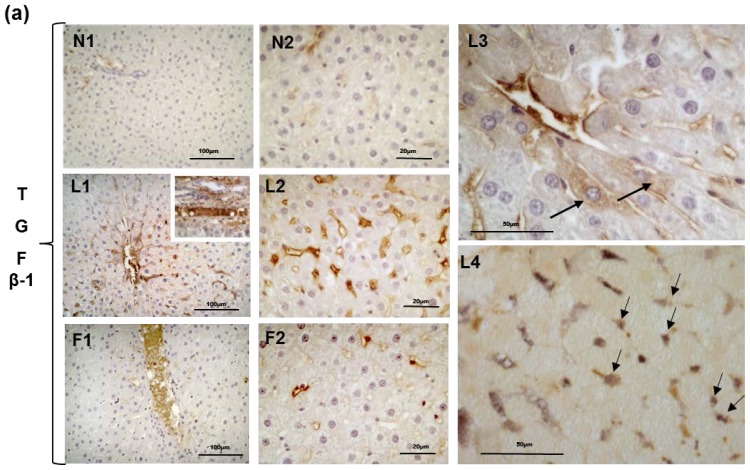

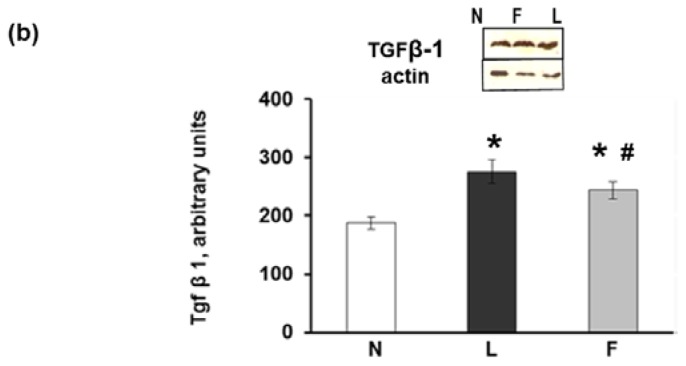
Immunostaining for TGFβ1 in sections from rats fed a control diet (N), high-lard diet (L) or high-fish oil diet (F). (**a**) The immunostaining for TGFβ1, in two different magnifications, was very weak and localised in the wall of a few blood vessels in the N livers. The number of TGFβ1-immunoreactive cells was increased in the L compared with the N rats, exhibiting a strong staining around the central vein. In L1-L2, the lumen of the blood vessel was filled with TGFα1-immunoreactive material (L1 insert). In L3, the cells lying along the wall of a blood vessel are heavily immunolabelled; in the parenchyma, two hepatocytes were weakly positive (arrows). L4 represents a double-immunostained section for TGFβ1 and CRBP1 indicating that most of the cells were immunoreactive to both antibodies. In the F rats, the TGFβ1-immunoreactive material was limited to the lumina of some blood vessels (F1-F2); (**b**) Representative Western blot analysis of TGFβ1. The relative levels in the N, L and F rat livers were densitometrically quantified and are expressed as the means ± SE. * *p* < 0.05 *vs.* N rats. ^#^ *p* < 0.05 compared with L rats

The activation of HSCs, which is the most critical step in the development of hepatic fibrosis, is mediated by cytokines and reactive oxygen species that are released by damaged hepatocytes and/or activated Kupffer cells and even HSCs themselves [48–50]. Although HSCs typically remain quiescent, they undergo transdifferentiation into myofibroblasts and become highly proliferative and fibrogenic in response to factors that promote liver injury [48–50]. The transdifferentiation of HSCs into contractile myofibroblasts was detected by analyzing the presence of α-actin of smooth muscle (α-SMA), which is a marker of myofibroblasts. Excluding the smooth muscle cells in portal areas, no reactivity for α-SMA was observed in the N or F rats, whereas in the L rats, the immunoreactivity for α-SMA was localised in cells in close proximity to hepatocytes in the sinusoid walls. These HSCs (which are trans-differentiating into myofibroblasts) were identifiable because they had not yet lost their lipid droplets (Figure 6c), L1–L3, arrows and insert). Western blot analysis of liver homogenates indicated the highest level of α-SMA in the L rats (Figure 6e). From our results, we can argue that although a diet high in fish oil induces a low degree of liver inflammation, it does not elicit marked injury at the hepatic level because the activation of HSCs is not evident (in contrast to rats fed a high-lard diet, which exhibit signs of the initial stages of HSC activation after six weeks). This suggestion is also confirmed by Western blot analysis and immunohistochemical analysis of TGFβ1 that demonstrate a lower TGFβ1 level in the F than in the L rats. TGFβ1, which is a cytokine that plays a pivotal role in the inflammatory response, may be expressed by both hepatocytes and mesenchymal cells. In the N rats, only weak immunostaining for TGFβ1 in the narrow areas of the parenchyma was present in and around the blood vessels (Figures 7a, N1–N2). In the L rats, the immunostaining for TGFβ1 was widely diffuse and very strong; in some cases, the lumen of the blood vessels was filled with immunoreactive material, and the perisinusoidal HSCs were abundantly labelled, as were some Kupffer cells. In addition, a small number of hepatocytes appeared moderately positive (Figure 7a), L1–L3). A double-immunostained section for CRBP1 and TGFβ1 indicated that most of the CRBP1-immunoreactive cells were simultaneously immunoreactive for TGFβ1 (Figure 7a, L4). The wall of numerous blood vessels and sinusoids were also strongly positive in F rats even if the immunoreactivity was less diffuse than that of the L rats (Figures 7a, F1–F2).

Western blot analysis of TGFβ1 in the L and F liver homogenates indicated higher levels than in the N homogenates, with L rats demonstrating the highest level of TGFβ1 (Figure 7b). Because TGFβ1 is related to fibrogenesis in the liver, the high-fish oil diet (in contrast to the high-lard diet) did not elicit the progression from steatosis to fibrosis after six weeks of diet treatment.

The beneficial effects of fish oil on hepatic steatosis may be mediated via adiponectin. It should also be noted that adiponectin regulates the activity of 5′-adenosine monophosphate activated protein kinase (AMPK), which is an important modulator of both glucose and lipid metabolism [51]. Thus, dietary fish oil, via increasing circulating adiponectin levels, can improve features of MetS such as IR, dyslipidemia, and hepatic steatosis.

## 3. Experimental Section

### 3.1. Materials

All chemicals used were of analytical grade and were purchased from Sigma (St. Louis, MO, USA). Mouse monoclonal anti-CD68 antibody (Ab), rabbit polyclonal anti-MCP1 (recombinant) Ab, rabbit polyclonal anti-glucose transporter Glut4 Ab and rabbit polyclonal anti-insulin receptor InsR Ab were obtained from Abcam (Cambridge, UK). Rabbit polyclonal anti-Galectin-3 Ab, goat polyclonal anti-TNFα Ab, rabbit polyclonal anti-TGFβ1 Ab, rabbit polyclonal anti-CRBP1 Ab, mouse monoclonal anti-α-SMA (smooth muscle α-actin) Ab, rabbit polyclonal anti-PPARγ and peroxidase-conjugated anti-rabbit or anti-mouse IgG were obtained from Santa Cruz Biotechnology (Santa Cruz, CA, USA). Biotinylated anti-goat IgG, streptavidin peroxidase complex and BSA were obtained from Vector (Burlingame, CA, USA). Anti-rabbit and anti-mouse antibodies used in the Polymer EnVision dual link kit were obtained from Dako. Polyvinyldifluoride membranes were obtained from Millipore Corporation (Bedford, MA, USA). Non-fat dry milk was obtained from Bio-Rad laboratories (Hercules, CA, USA). The enhanced chemiluminescence kit was obtained from Amersham (Little Chalfont, Buckinghamshire, UK).

### 3.2. Animals and Diets

Male Wistar rats, age 60 days (Charles River Italia, Calco, Como, Italy), were caged separately in a temperature-controlled room at 24 °C with a 12 h light-dark cycle.

At the start of the study, the rats were divided into three groups (including eight rats each) with a similar mean body weight (approximately 400 g) and with the body weights normally distributed within each group. The first group (referred to as N) received a standard diet (10.6% fat J/J); the second group (referred to as L) received a high-fat diet rich in lard (40% fat J/J); the third group (referred to as F) received a high-fat diet rich in fish oil (40% fat J/J). The animals were fed *ad libitum* and the period of treatment lasted six weeks. The composition of the three diets is shown in Table 2. The two high-fat diets were formulated to differ from the standard low-fat diet in the fat and carbohydrate contribution to the energy value but to be identical in terms of proteins, vitamins, minerals and fibres, as previously reported [52]. Throughout the experimental period, body weight and food intake were monitored daily. Food spilled was carefully collected and accounted for in the food intake calculations.

The experimental design was repeated three times (different rats used each time) for all required measurements. In particular, groups of rats were fasted overnight to determine basal glucose and serum insulin levels. To detect insulin-dependent Glut4 and insulin receptor immunoreactivity, groups of rats (named insulin-injected (ii) rats) that had fasted for five h were euthanized 15 min after an i.p. injection of insulin (homolog rapid-acting, 10 units/kg body wt; Novartis, Basel, Switzerland), as previously reported [53]. All animals received humane care according to the criteria set by the National Institutes of Health.

At the end of the experimental period, the animals were anaesthetized via an injection of chloral hydrate (40 mg/100 g body weight, i.p.), and blood was collected via the inferior cava vein. Immediately after blood collection, the liver, skeletal muscle (*gastrocnemius*) and epididymal white adipose tissue (eWAT) were either immediately fixed and processed for immunohistochemistry analysis or immediately frozen in liquid nitrogen and stored at −80 °C for subsequent processing.

### 3.3. Determination of Serum Parameters

Glucose concentrations were determined using a glucose monitor (BRIO, Ascensia, NY, USA). Serum leptin, adiponectin, MCP1, TNFα and insulin concentrations were measured using ELISA kits (B-Bridge International Inc. for leptin and adiponectin; Thermo Scientific, Rockford, IL, USA for TNFα and MCP1; and Mercodia, Uppsala, Sweden for insulin).

### 3.4. Histology

Small pieces of eWAT, gastrocnemius skeletal muscle and liver were fixed with the following fixatives: Carnoy’s solution, Bouin’s fluid, neutral buffered formaldehyde (4%) and 2.5% glutaraldehyde in Millonig’s buffer. Some fixed tissue pieces were embedded in Paraplast and sectioned to 4–5 μm, whereas others were embedded in EPON 812 and sectioned to 1–3 μm to achieve semi-thin sections. The sections were stained with haematoxylin and eosin (H&E) and toluidine blue and examined under a Zeiss Axioskop light microscope. Digital images were acquired using a TV camera attached to a Zeiss Axioskop microscope equipped for bright field and fluorescence microscopy. The histochemical PAS (periodic acid and Schiff reactive) stain with or without diastase digestion was performed in liver sections to detect glycogen depots. The PAS positive material was stained magenta.

### 3.5. Determination of Adipocyte Size Using Computer Image Analysis

The digital image processing software Zeiss AxioVision (Zeiss, Göttingen, Germany) was used to assess the diameter of the adipocyte. The adipocyte diameters were interactively measured on at least 100 cells/animal (eight animals/group).

### 3.6. Immunohistochemistry

Serial sections were immunostained using Labeled Streptavidin-Biotin (LSAB) and the polymer Envision dual link kit. After antigen retrieval and endogenous peroxidase quenching, the sections were incubated with anti-CD68 (diluted 1:200), anti-Galectin-3 (Gal3) (1:200), anti-MCP1 (1:100), anti-TNFα, anti-TGFβ1 (1:100), anti-CRBP1 (1:100), anti-α-SMA (smooth muscle α-actin) (1:100), anti-Glut4 (1:200), anti-insulin receptor (InsR) (1:200) and anti-PPARγ (1:400). The diluted Abs were incubated overnight at 4 °C. The immunostaining was performed using DAB, AEC and 4-chloro-1-naphthol as chromogens. Endogenous Fc receptors were blocked with 20% FCS (foetal calf serum) in PBS. The following control procedures were performed to evaluate the specificity of the reagents: (1) omission of the primary antiserum and incubation of the sections with either non-immune serum (1:10 and 1:20) or BSA; (2) absorption of the primary antiserum with its specific peptide (10 nmol/mL of optimally diluted antiserum) for 24 h at 4 °C. When specific peptides were used, the staining was abolished.

### 3.7. Preparation of Skeletal Muscle and Liver Extract

Pieces of the skeletal muscle and liver of the N, L and F rats were rinsed with PBS, removed, weighed, minced, pooled and homogenized (10% *w*/*v*) using a glass potter homogeniser in a lysis buffer. Skeletal muscle and liver homogenates in RIPA buffer (1% Nonidet P-40, 0.5% sodium dodecyl sulphate (SDS), 100 nM sodium fluoride, 2 mM tetrasodium pyrophosphate, 2 mM orthovanadate and 5 mM EDTA in PBS) were freshly added with protease inhibitors (1 mM phenylmethylsulfonyl fluoride, 10 μg/mL aprotinin, 10 μg/mL leupeptin and 10 μg/mL pepstatin), filtered through three layers of cheesecloth and centrifuged at 14,000 × *g* at 4 °C for 30 min. The supernatant was collected and stored at −80 °C until further analysis. All procedures were performed at 4 °C.

### 3.8. Lipid and TNF-α Content

Lipid content was determined using the method of Folch *et al.* [40] in the liver and skeletal muscle. The TNF-α concentration was assessed in the skeletal muscle and liver homogenates using ELISA (Thermo Scientific, Rockford, IL, USA).

### 3.9. Electrophoresis and Western Blot Analysis

The protein concentration was determined using the Coomassie Protein Assay Kit (Pierce Biotechnology, Rockford, IL, USA). Samples of liver extract were electrophoresed on 8–10 or 15% SDS-PAGE under denaturing conditions, as described by Laemmli [54]. After electrophoresis, the proteins were transferred onto a polyvinyldifluoride membrane according to Towbin *et al.* [55]. The antibodies raised against CD68, Gal3, MCP1, α-SMA, TGFβ1 and β-actin were used as primary antibodies. Peroxidase-conjugated anti-rabbit or anti-mouse IgG (1:2000) in TTBS containing 5% non-fat dry milk were used as secondary antibodies with 1-h incubation at RT. The molecular markers were obtained from Sigma. The immunoreactive bands were visualized using enhanced chemiluminescence, according to the manufacturer’s instructions. The relative densities of the immunoreactive bands were determined and normalized with respect to β-actin using a semiquantitative densitometric analysis (Kodak ID Image Analysis Software Rochester, NY, USA).

### 3.10. Data Analysis

Data are expressed as the means ± standard errors in the descriptive text and figures. Differences between groups were compared using ANOVA followed by the Newman-Keuls test to correct for multiple comparisons. Differences were considered significant at *p* < 0.05. All analyses were performed using GraphPad Prism 4 (GraphPad Software, San Diego, CA, USA).

## 4. Conclusions

By comparing the effect of a high-lard and a high-fish oil diet, our study demonstrates that the quality of the dietary fat in high-fat chronic overfeeding differentially affects the inflammatory intra-organ pathways, morphological features and inter-organ cross-talk through their different effects on serum adipokine levels and systemic IR. A high-fish oil diet attenuates obesity and related systemic and peripheral effects such as insulin resistance, the expression of inflammatory markers and the stimulation of inflammatory cells. Interestingly, our results indicate that a high-fish oil diet is not completely anti-inflammatory, as is typically reported for ω-3 PUFA supplementation, which was likely due to the high dose of fat present in our experimental diet. However, a high-fish oil diet does not induce marked liver injury and prevents both HSC activation and progression to fibrosis.

## Figures and Tables

**Table 1.** Body weight gain, energy intake and feeding efficiency in rats fed a normal, high-lard and high-fish oil diet.

	N	L	F
Body-weight gain, g/week	16.8 ± 1.0	29.7 ± 3.1 *	21.5 ± 1.9 #
Food intake, g/week	136.4 ± 10.1	167.5 ± 9.2 *	168.3 ± 8.3 *
Feeding efficiency, %	12.3 ± 1.1	17.7 ± 1.5 *	12.8 ± 1.0 #
Retroperitoneal WAT, g	7.05 ± 0.31	16.27 ± 0.73 *	9.8 ± 0.51 *#
Epididymal WAT, g	7.97 ± 0.28	14.6 ± 1.6 *	11.52 ± 0.61 *#

The data represent the means ± SE for 8 rats in each experimental group;

* *p* < 0.05 compared with the N rats;

# *p* < 0.05 compared with the L rats; N = rats fed a normal low-fat diet; L = rats fed a high-lard diet; and F = rats fed a high-fish oil diet.

**Table 2.** Diet compositions.

Component	Control Diet	High-fat diet	

		High-Lard g/100 g diet	High-Fish Oil g/100 g diet
Standard feed g	100	51.03	51.03
Casein a g	-	9.25	9.25
Lard g	-	21.8	-
Fish oil b g	-	-	21.8
Sunflower oil g	-	1.24	1.24
AIN 76 Mineral mix c g	-	1.46	1.46
AIN 76 Vitamin mix d g	-	0.42	0.42
Choline bitartrate g	-	0.08	0.08
Methionine g	-	0.12	0.12
Energy density, kJ/g diet	15.88	20.00	20.00
Energy (J/100 J)			
Protein%	29	29	29
Lipid%	10.6	40	40
Carbohydrate%	60.4	31	31

a Purified high-nitrogen casein containing 88% protein;

b Fish oil= The fish oil used was cod liver oil (New.Fa.Dem. srl, Giugliano, Naples, Italy) containing vitamin A (50–500 UI/g; 15–150 μg) and vitamin D3 (50 U.I./g; 1.3 μg); EPA ≈ 722 mg/Kg body weight/die and DHA ≈ 1153 mg/kg body weight/die;

c American Institute of Nutrition (1977);

d American Institute of Nutrition (1980).
